# Serine-71 Phosphorylation of Rac1 Modulates Downstream Signaling

**DOI:** 10.1371/journal.pone.0044358

**Published:** 2012-09-10

**Authors:** Janett Schwarz, Julia Proff, Anika Hävemeier, Markus Ladwein, Klemens Rottner, Britta Barlag, Andreas Pich, Helma Tatge, Ingo Just, Ralf Gerhard

**Affiliations:** 1 Department of Toxicology, Hannover Medical School, Hannover, Germany; 2 Department of Virology, Hannover Medical School, Hannover, Germany; 3 Cytoskeleton Dynamics Group, Helmholtz Centre for Infection Research, Braunschweig, Germany; 4 Institut für Genetik, Rheinische Friederich-Wilhelms-Universität, Bonn, Germany; Institute of Developmental Biology and Cancer Research, France

## Abstract

The Rho GTPases Rac1 and Cdc42 regulate a variety of cellular functions by signaling to different signal pathways. It is believed that the presence of a specific effector at the location of GTPase activation determines the route of downstream signaling. We previously reported about EGF-induced Ser-71 phosphorylation of Rac1/Cdc42. By using the phosphomimetic S71E-mutants of Rac1 and Cdc42 we investigated the impact of Ser-71 phosphorylation on binding to selected effector proteins. Binding of the constitutively active (Q61L) variants of Rac1 and Cdc42 to their specific interaction partners Sra-1 and N-WASP, respectively, as well as to their common effector protein PAK was abrogated when Ser-71 was exchanged to glutamate as phosphomimetic substitution. Interaction with their common effector proteins IQGAP1/2/3 or MRCK alpha was, however, hardly affected. This ambivalent behaviour was obvious in functional assays. In contrast to Rac1 Q61L, phosphomimetic Rac1 Q61L/S71E was not able to induce increased membrane ruffling. Instead, Rac1 Q61L/S71E allowed filopodia formation, which is in accordance with abrogation of the dominant Sra-1/Wave signalling pathway. In addition, in contrast to Rac1 transfected cells Rac1 S71E failed to activate PAK1/2. On the other hand, Rac1 Q61L/S71E was as effective in activation of NF-kappaB as Rac1 Q61L, illustrating positive signal transduction of phosphorylated Rac1. Together, these data suggest that phosphorylation of Rac1 and Cdc42 at serine-71 represents a reversible mechanism to shift specificity of GTPase/effector coupling, and to preferentially address selected downstream pathways.

## Introduction

The small Rho GTPases are monomeric GTP-binding proteins that play a role in a variety of cellular processes that depend on the actin cytoskeleton, such as morphogenesis, endocytosis, phagocytosis, cytokinesis and migration. They act as nucleotide-dependent switches cycling between an active, GTP-bound state and an inactive, GDP-bound state. In their GTP-bound state, they interact with downstream effectors to initiate downstream signaling. The binding of Rho GTPases to their effector proteins occurs by distinct binding motifs. Most effector proteins harbor the common Cdc42/Rac1 interactive binding motif (CRIB), which is present in the p21-activated kinase (PAK) and the Wiskott-Aldrich syndrome protein (WASP) [1]. The myotonic dystrophy related Cdc42 kinase (MRCK) also contains a CRIB-related binding domain (PBD) [2]. In addition Rac1/Cdc42 bind to the RasGAP-homology domain which is present in IQGAP [3]. The specifically Rac1-associated protein (Sra-1), a subunit of the pentameric WAVE complex, does not harbor a CRIB-motif and can directly bind to Rac1 but not to Cdc42 [4]. Each of these effectors contributes to the cytoskeletal reorganization downstream of Rac1 and Cdc42, driving the formation of a diverse array of actin structures, e. g. membrane ruffles and lamellipodia. Formation of these structures is stimulated by Rac1, whereas Cdc42 induces the formation of filopodia and microspikes [5]. Additionally, Rho GTPases influence gene expression by regulating signaling pathways involving the transcription factor NF-κB, c-Jun N-terminal kinase (JNK), and p38 mitogen-activated protein kinase, and they drive G1 cell cycle progression, apoptosis and cell transformation. Activation of Rho GTPases is regulated by three types of proteins: The guanine nucleotide dissociation inhibitors (GDIs), the guanine nucleotide exchange factors (GEFs), and GTPase activating proteins (GAPs) [6]. The GDIs stabilize the inactive, GDP-bound form of the Rho GTPases in the cytosol (Rho-GDI complex) and thus prevent association with the membrane [7]. The GEFs catalyze the exchange of GDP for GTP, which is thought to be coordinated with membrane targeting of Rho GTPases [8]. The GAPs stimulate the intrinsic GTPase activity and convert the GTP-bound form of Rho GTPases to the inactive, GDP-bound form [9].

Phosphorylation of Rho GTPases is an additional mechanism to modulate the activity of these proteins, mainly leading to their functional inactivation. Phosphorylation was first described for RhoA, which can be phosphorylated by PKA/PKG at Ser-188 [10]–[12] resulting in cytosolic relocalization due to an increased binding to Rho-GDI. Cdc42 also harbors a PKA phosphorylation site at Ser-185 [11]. Additionally, EGF treatment of cells can induce the phosphorylation of Cdc42 at tyrosine-64 by Src kinase. This specific phosphorylation does not affect interaction with effector proteins but leads to increased interaction with Rho-GDI [13]. Very recently, the same was reported for Rac1, where phosphorylation of Tyr-64 affects interaction with PAK and cell spreading as shown by transfection experiments with the phosphomimetic mutant Rac1 Y64D [14]. Rac1 can also be phosphorylated by the Akt kinase at Ser-71, which is embedded in the consensus sequence ^64^yd*R*IRpl*SY*p^73^. Most Rho GTPases, e. g. Cdc42 and RhoA/B/C/G share this sequence. The Ser-71-phosphorylation of Rac1 results in reduced GTP-binding without affecting GTPase activity [15]–[16]. Ser-71-phosphorylated Rac1/Cdc42 appear to be in their active conformation according to pull down assays with PAK CRIB-domain and Rho-GDI. RhoE can be phosphorylated by ROCK I at Ser-11 resulting in the cytosolic relocalization and an increased stability of the GTPase [17]. In summary, phosphorylation is not the key event in activation/inactivation of Rho GTPases, but it predominantly modulates affinity to GDI and subcellular localization of the GTPase, thereby negatively affecting its activity. In the present study we show that phosphorylation of Rac1 and Cdc42 at Ser-71, in addition, modulates downstream signaling by inhibiting interaction with some effectors but allowing interaction with others. This is the first description of the functional regulation of effector coupling by phosphorylation of Rac1/Cdc42 at Ser-71 as an additional mechanism to specify downstream signaling of the activated GTPases.

## Results

### Ser-71-Phosphorylation of Rac1 Induces a Cdc42-like Phenotype

**Figure 1:**
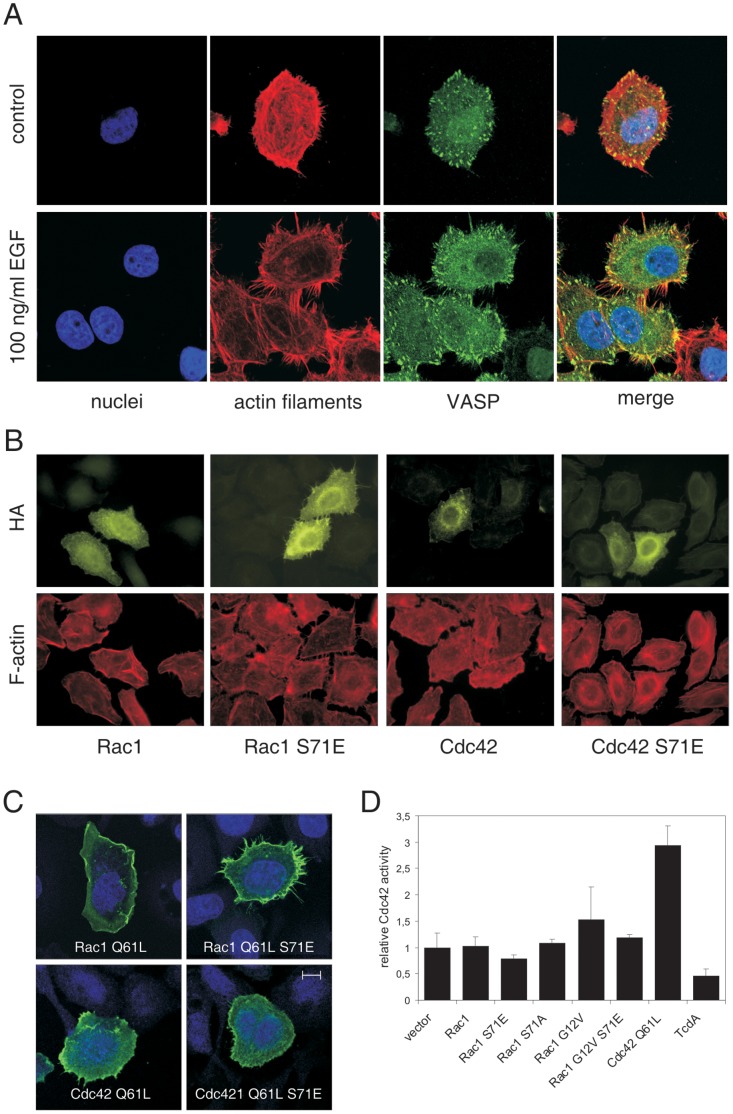
Phosphomimetic Rac1 S71E induces filopodia formation. A) Treatment with 100 ng/ml EGF for 2 h induces pronounced formation of filopodia. Cells were stained for nuclei (DAPI, blue), actin cytoskeleton (rhodamin-phalloidin, red), and VASP (Alexa-488, green). B) HEp2 cells transfected with HA-tagged Rac1, Rac1 S71E, Cdc42, and Cdc42 S71E. Expression of GTPases was visualized by HA-staining, the actin cytoskeleton was stained with rhodamin-phalloidin. Only Rac1 S71E induced morphotype that is comparable with EGF-induced alterations. C) Phenotypes of HEp2 cells transfected with HA-tagged constitutive active mutants of Rac1 and Cdc42 as well as their phosphomimetic mutants S71E. Constitutively active (Q61L) Rac1 induced membrane ruffling whereas Rac1 S71E induced formation of filopodia. Filopodia formation is less pronounced in Cdc42 Q61L and Cdc42 Q61L/S71E transfected cells. Stained are nuclei (blue) and HA-tag (green); bar represents 10 µm. D) Active, GTP-bound form of Cdc42 was determined by G-LISA 24 h post transfection with constructs as indicated. Cdc42 Q61L was used for transfection experiments as positive control for experimental setup. Additionally, *C. difficile* toxin A (TcdA) was used as negative control for inactivation of Cdc42. The bar chart shows mean values ± SD of three (for TcdA) or four separate experiments.

Phosphorylation of Rac1/Cdc42 induces a specific phenotype of cells. Treatment of cells with the epidermal growth factor (EGF) induces Rac1/Cdc42 phosphorylation which is accompanied by the formation of filopodia [16]. EGF-induced filopodia formation is illustrated in Fig. 1A, showing staining of the actin cytoskeleton and the localization of the vasodilator-stimulated phosphoprotein (VASP). VASP was visualized as marker for filopodia [18] to dissect these structures from retraction fibers. Morphological effects of Rac1 and Cdc42 as well as their S71E mutants are shown in Fig. 1B. Only cells transfected with Rac1 S71E showed increased formation of filopodia, whereas Rac1, Cdc42, and Cdc42S71E transfected cells showed phenotype of non-transfected controls. The effect was also obvious when Rac1 S71E with constitutive active (Q61L) background was used. As shown in Fig. 1C, Rac1 Q61L induced formation membrane ruffles, whereas Rac1 S71E Q61L strongly induced formation of filopodia. Rac1 S71E Q61L-induced filopodia were compared with those induced by constitutive active Cdc42 (Q61L). Formation of filopodia by Cdc42 Q61L was less pronounced than in Rac1 Q61L/S71E transfected cells. The phenotype of cells expressing the double mutant Cdc42 Q61L/S71E corresponded to the Cdc42 Q61L phenotype with more microspike like structures. It is of importance to investigate morphological effects of the Q61L mutants of Rac1/Rac1 S71E and Cdc42/Cdc42 S71E because these mutants were used later on for pull down experiments to characterize effector coupling.

To investigate the mechanism leading to filopodia formation in more detail, we checked whether overexpression of Rac1 S71 somehow induces activation of Cdc42. Neither overexpression of wildtype Rac1 nor of Rac1 S71E nor the control mutant Rac1 S71A induced activation of Cdc42 (Fig. 1D). The constitutive active mutant Rac1 G12V showed weak but not significant effect on Cdc42 activation. In Rac1 G12V/S71E transfected cells, however, no sign of Cdc42 activation was observed at all. Transfection experiments with Cdc42 Q61L were performed as positive control for experimental setup. *C. difficile* TcdA, which catalyzes inactivation of Rho GTPases including Cdc42 by mono-glucosylation served as negative control. These results indicate that the Rac1 S71E-induced filopodial phenotype is not caused by concomitant activation of Cdc42. It is well established that filopodia formation can be enforced by suppression of Rac1 signaling, either directly through microinjection of mixtures of active Cdc42 and inactive Rac1 [5], or more indirectly when interfering with Rac effector function [19]. We suppose this principal as reason for the Rac1 S71E-induced changes in cell morphology.

### Phosphorylation of Rac1 and Cdc42 at Ser-71 Modulates Effector Binding

**Figure 2:**
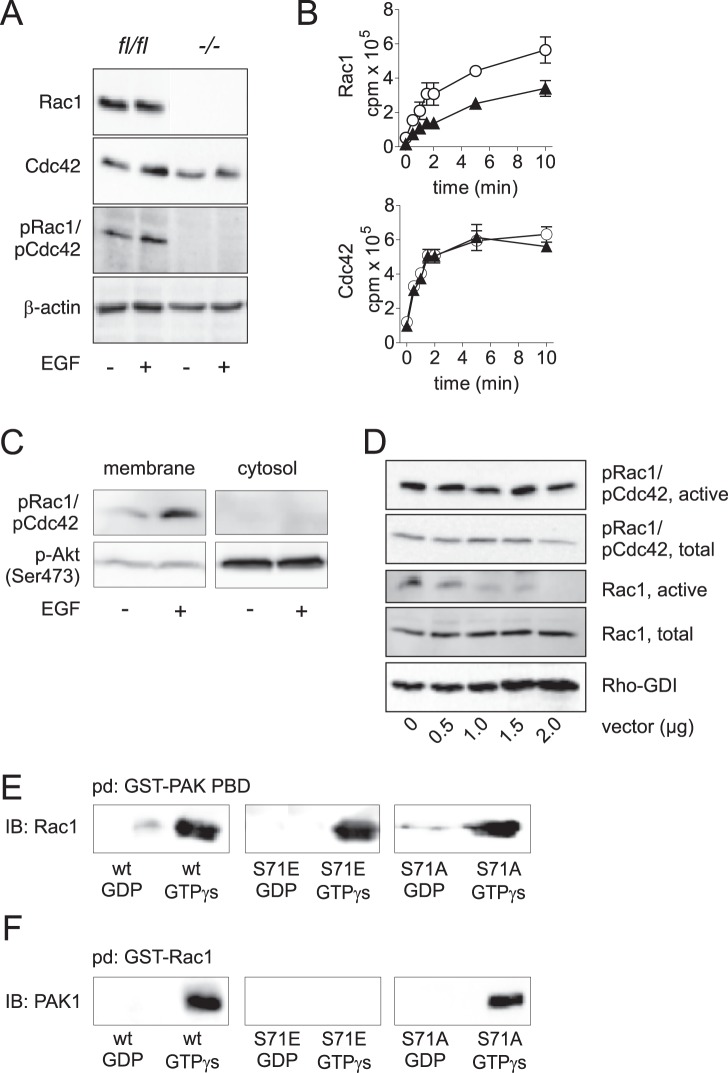
Activity status of Rac1 S71E and Cdc42 S71E. A) Cell lysates of *Rac1^fl/fl^* and *rac1* ^−/−^ mouse fibroblasts were analyzed for Ser-71 phosphorylation of Rac1 and Cdc42 after treatment with 100 ng/ml EGF for 2 h by immunoblot using anti-pRac1/pCdc42 (Ser71) antibody. Lack of specific signal in *rac1* ^−/−^ cells strongly suggested specific phosphorylation of Rac1 and no detectable phosphorylation of Cdc42. Immunoblot is representative for three separate experiments B) GTP-binding of phosphomimetic (▴) Rac1 S71E and Cdc42 S71E in comparison with wild-type (○) Rac1 and Cdc42 was analyzed by a [γ-^32^P]-GTP-binding assay. The diagrams show mean values ± SD of three separate experiments. C) Intracellular localization of pRac1/pCdc42 (Ser71). Phosphorylated Rac1/Cdc42 is exclusively present in the membrane fraction of cells. D) Pull down assay of pRac1/pCdc42 and Rac1 using PAK-PBD after overexpression of Rho-GDI. Total Rac, total pRac1/pCdc42, and expression of Rho GDI were checked by immunoblot of whole cell lysate (lower panel). E) Pull down assay in a recombinant system showed nucleotide dependent binding of wild-type Rac1, Rac1 S71E and Rac1 S71A to the PAK-p21 binding domain. E) The nucleotide-dependent binding of Rac1, Rac1 S71E and Rac1 S71A to full length PAK1 as determined by pull down experiments with HEp2 cell lysates using GTPases as bait.

Unfortunately, it is not possible to dissect between Ser-71 phosphorylation of Rac1 and Cdc42. To date there is no antibody that differentiates between pSer-71-Rac1 and pSer-71-Cdc42. We therefore took advantage of murine fibroblasts lacking the Rac1 gene (*rac1* ^−/−^) to check for pSer-71 staining of Rac1 and Cdc42. As shown in Fig. 2A, *rac1* ^−/−^ fibroblasts do not express detectable levels of Rac1. Cdc42 expression, however, was comparable to the parental cell line *rac1^fl/fl^* fibroblasts. Beta-actin served as loading control. In contrast to control fibroblasts, Rac1-deficient cells were completely devoid of pSer-71-Rac1 and pSer-71-Cdc42 staining, even after treatment of cells with 100 ng/ml EGF. This strongly argues that EGF mainly drives phosphorylation of Rac1, at least in fibroblasts. With respect to this finding, we assumed that the observed effects in the present study are mainly due to Rac1 phosphorylation.

To better understand the outcome of Ser-71 phosphorylation at the molecular level, we explored nucleotide-binding of the wild type forms of Rac1 and Cdc42 in comparison with their phosphomimetic mutants (Fig. 2B). GTP-binding of the phosphomimetic Rac1 mutant (S71E) was markedly reduced compared to wild-type Rac1 (upper panel). This data is in line with results published by Kwon et al [15], who also found reduced GTP-binding of phosphorylated Rac1. We previously reported that Rac1 S71E is able to bind GTP [16], and it is noteworthy that GTP-binding of the phosphomimetic mutant was only reduced to some extent, but not abolished. This finding is in accordance with previously reported pull down assays showing active conformation of Ser-71 phosphorylated Rac1. Mutation of serine to alanine (S71A) did not alter the GTP-binding, indicating a specific effect of the phosphomimetic mutation (data not shown). In contrast to Rac1, no difference in GTP-binding between Cdc42 wild-type and phosphomimetic Cdc42 was observed (Fig.2B, lower panel). We also provided indirect evidence of active state of Ser-71 phosphorylated Rac1: Immunoblot analyses showed that constitutive and EGF-induced pRac1/pCdc42 exclusively locates at the membranes (<100,000×*g* fraction) of HEp2 cells (Fig. 2C). No signal was detected within the cytosol (>100,000×*g* fraction). The majority of activated Akt kinase was detected within the cytosol but also to some extend within the membrane fraction. Furthermore, Overexpression of Rho-GDI in HEp2 cells did not reduce level of active pRac1 as tested in pull down assay with PAK p21 binding domain (PAK-PBD) (Fig. 2D). In contrast, non-phosphorylated active Rac1 was reduced with increasing amount of overexpressed Rho-GDI. Previous studies showed binding of pRac1 (S71) to the PAK-PBD [16]. We thus initially analyzed binding of Rac1 S71E to PAK1 to show specificity of precipitation experiments. By using immobilized PAK1-PBD as bait in a recombinant system we were able to show specific and nucleotide-dependent interaction with Rac1 and Rac1 S71E (Fig. 2E). Unspecific binding of Rac1 S71E was excluded by comparison with an alternative mutant, where S71 was exchanged to alanine (S71A). Rac1 S71A showed identical binding to PAK-PBD as wild-type Rac1. When performing the contrary experiment, where Rac1, Rac1 S71E and Rac1 S71A were used as bait, surprisingly only wild-type and the control mutant S71A of Rac1 were able to bind and to precipitate full length PAK1 from HEp2 cell lysates (Fig. 2F). Rac1 S71E did not interact with PAK1, neither in its active (GTPγS-loaded) nor inactive (GDP-bound) form. This is interesting because similar results were published by Matos et al, who showed that Rac1b, a splice variant of Rac1 with 19 additional amino acids following switch 2 region, also interacts with the PAK-PBD domain but not with full length PAK1 [20]. This result emphasizes the importance of the switch 2 region for effector interaction.

**Figure 3:**
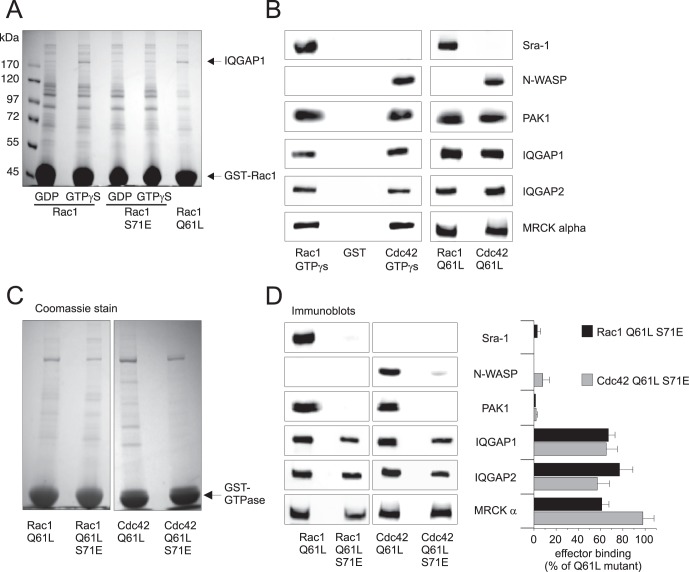
GTPase-specific binding to their effectors. A) Coomassie-stained SDS-gel of precipitates of pull down experiments from HEp2 cell lysate. Arrows indicate GST-Rac1/−Rac1 S71E (48 kDa) used as bait and a coprecipitated 190 kDa protein that was identified as IQGAP1 by MALDI-TOF/TOF analysis. B) The interaction of active, GTP-bound Rac1/Cdc42 and their active forms (Q61L) with specific effectors was analyzed by immunoblot analyses of precipitates from pull down assays. Non-specific binding was tested by GST-loaded glutathione beads as control. C) Representative input control of pull down analyses using constitutively active (Q61L) mutants of Rac1 and Cdc42 and their S71E mutants. D) Representative immunoblots of pull down precipitates showing the interaction of constitutively active Rac1 and Cdc42 and constitutively active S71E mutants with their effector proteins. Rac1 Q61L/S71E and Cdc42 Q61L/S71E did hardly bind to their specific effectors Sra-1 and N-WASP, respectively and to their common effector protein PAK1. Both phosphomimetic GTPases, however bound to their common effectors IQGAP and MRCK alpha, although to a lesser extent. The bars show the arithmetic mean value ± SD of densitometrical evaluation of 3 independent experiments.

Based on these findings, we performed further pull down experiments from HEp2 cell lysates where GST-fusion proteins of wild type and mutant (S71E) Rac1 and Cdc42 (data not shown) were used as baits to identify possible interacting proteins. Figure 3A shows a Coomassie-stained SDS-gel of precipitates from HEp2 cell lysates where the nucleotide-dependent binding of interacting proteins was analyzed. An approximately 190 kDa-sized band was visible that showed increased binding to GTP-loaded Rac1 and Rac1 S71E. Constitutively active Rac1 Q61L was additionally used as positive control. Mass spectrometry analysis identified IQGAP1 as interacting protein within this range of about 190 kDa. Since it was not clear whether the prominent coomassie-stained band definitely reflects IQGAP, all further precipitates were analyzed by immunoblots to specifically detect IQGAP and additional effector proteins such as PAK1, N-WASP, Sra-1 and MRCK alpha.

Therefore, the specificity of binding to effector proteins was checked by using GTPγS-loaded wild-type GTPases. Additionally, constitutive active (Q61L) mutants of Rac1 and Cdc42 were compared with respect to their specificity. Comparison of GTPγS loaded GTPases with constitutive active ones was done to evaluate the Q61L mutants for further experiments. In fact, GTPγS-loaded Rac1 and the constitutively active (Q61L) mutant interacted with the Rac1-specific effector protein Sra-1 but not with the Cdc42-specific effector N-WASP (Fig. 3B). In contrast, active Cdc42 interacted with N-WASP but not with Sra-1. Both GTPases bound to their common effectors PAK1, IQGAP1 and 2 and the alpha form of MRCK. Unspecific precipitation of effectors was excluded by using GST-bound glutathione beads as control. The constitutively active (Q61L) mutants of Rac1 S71E and Cdc42 S71E were further on used for analysis of effector interaction in comparison with wild-type GTPases. The Q61L/S71E double mutants of Rac1 and Cdc42 were chosen to overcome incomplete GTPγS-loading of either GTPase. Double mutants allowed semi-quantification of the phosphomimetic mutants in comparison with the constitutively active wild-type GTPases and warranted exclusion of false negative results.

Pull down experiments with constitutively active GTPases were performed in triplicate. Figure 3C shows representative input controls of GST-GTPases and Fig. 3D shows representative immunoblots of precipitates from pull downs: The phosphorylation of Rac1 and Cdc42 at serine-71 as represented by the S71E mutants fully abrogated binding of Cdc42 to N-WASP (the Cdc42 effector), of Rac1 to Sra-1 (the Rac1 effector) and of both to their common effector PAK 1. The interaction with the isoform 1 and 2 of IQGAP and the alpha isoform of MRCK was only reduced to some extent or even unchanged. Since cell lysates were adjusted to same protein concentrations, MRCK also serves as loading control. Densitometrical evaluation of immunoblots (Fig. 3D left panel) revealed that binding of Rac1 Q61L/S71E to IQGAP1, IQGAP2 and MRCK alpha was reduced to about 60–70% of Rac1 Q61L binding. Cdc42 S71E showed an approximately 30% reduced binding to both IQGAP isoforms. The interaction of Cdc42 Q61L with MRCK alpha was not affected by exchange of Ser-71 to Glu-71. In conclusion, these data clearly show that phosphorylation of Rac1 and Cdc42 interferes with the binding to their GTPase-specific effectors Sra-1 and N-WASP as well as to their common effector PAK1. In contrast, the binding to IQGAP1/2 and MRCK alpha is only reduced or not even affected. The effect of the serine-71 phosphorylation on the binding to their effectors was slightly different between both GTPases. Whereas Rac1 showed the strongest binding with IQGAP2, Cdc42 showed strongest interaction with MRCK alpha. With respect to the consequences on the molecular level phosphorylation on Ser-71 of each GTPase seems to minimize differences of effector binding between Rac1 and Cdc42.

### Phosphorylation-induced Loss of Function of Rac1

**Figure 4:**
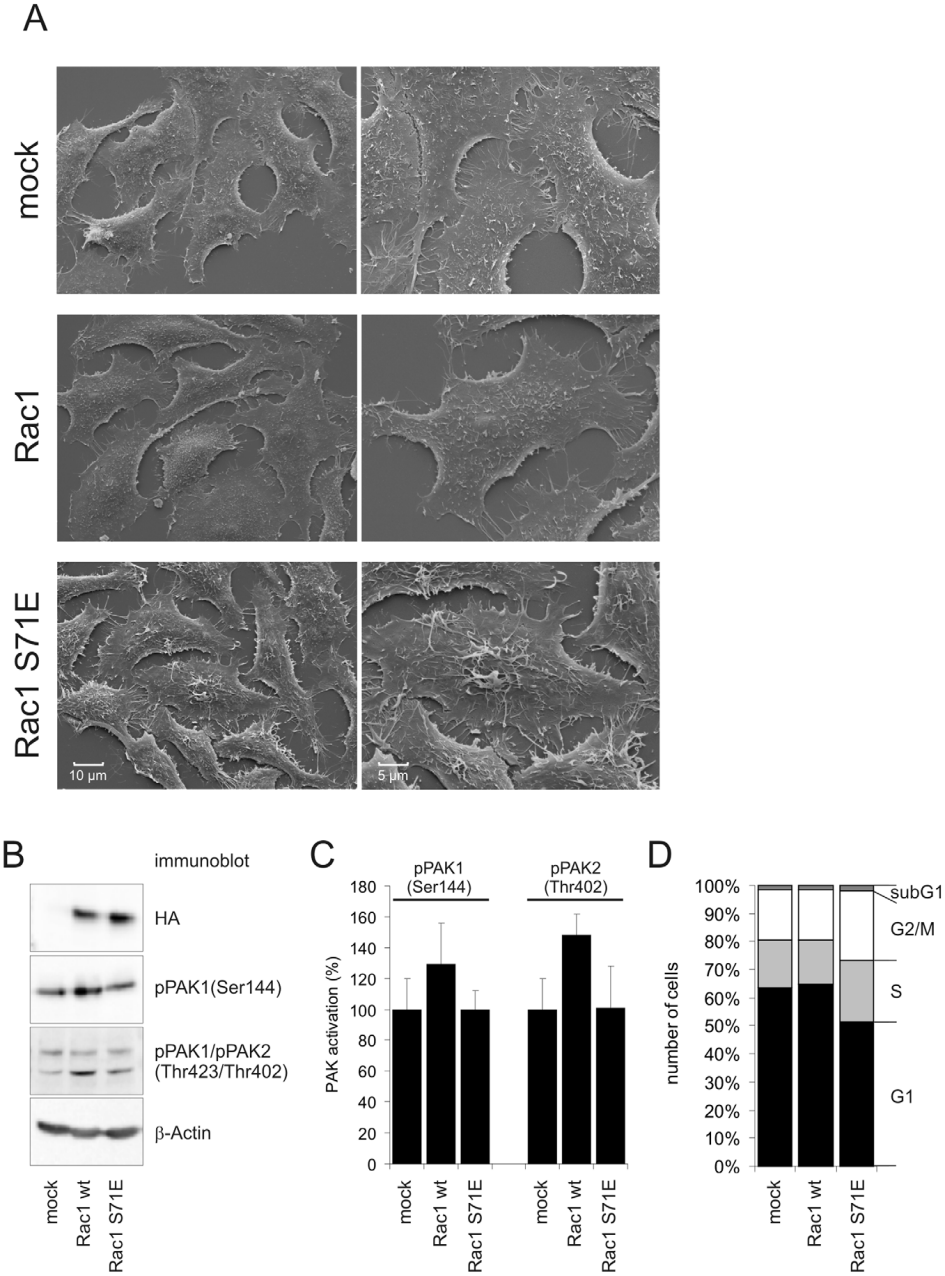
Activation of PAK by Rac1 and Rac1 S71E. The effect of Rac1 and Rac1 S71E on PAK phosphorylation was shown in HEp2 cells stably expressing either GTPase. A) A brief characterization of these stable transfected cell lines was done by scanning electron microscopy showing surface topology of the cells. B) Immunoblot analysis revealed comparable ectopic expression of HA-tagged Rac1 and Rac1 S71E and concomitant Ser-144 phosphorylation of PAK1 and Thr-402 phosphorylation of PAK2. C) densitometrical evaluation of three separate Immunoblots showing phosphorylation of PAK 1/2. Shown are mean values ± SD. D) Propidium iodide staining of stable cell lines indicates populations of cells with 2n (G1 phase) or 4n (G2/M phase) set of chromosomes. Shown are percentages of cells within different cell cycle phases (mean values of five separate experiments).

Results shown in figure 2 and 3 show, that Ser-71 phosphorylation interfered with binding of Rac1 to full length PAK1. We performed immunoblot analyses to check PAK activation by Rac1 and Rac1 S71E and possible functional consequences. To ensure homogenous expression of GTPases we generated stable transfected HEp2 cells expressing Rac1 and Rac1 S71E. A brief characterization of these cell lines is shown in figure 4. Scanning electron microscopy graphs reveal different surface topology of Rac1 S71E expressing cells compared with mock transfected cells or cells expressing wild type Rac1 (Fig. 4A). Representative figures show filopodia like protrusions in Rac1 S71E cells whereas cell surface of Rac1 cells is as smooth as in mock transfected cells. Expression of HA-tagged GTPases is shown in immunoblot (Fig. 4B). The Rac1 expressing cell line showed increased Ser-144 phosphorylation of PAK1 and Thr-402 phosphorylation of PAK2. In contrast, expression of Rac1 S71E failed to induce PAK1 or PAK2 phosphorylation. Densitometrical evaluation of three separate immunoblots is shown in Fig.4C. These data are in line with the precipitation experiment where Rac1 S71E was shown not to interact with full length PAK1. Additionally, compared to mock or wild type Rac1 transfected cells proliferation of Rac1 S71E expressing cells is reduced, and flow cytometry of cells stained with propidium iodide enumerated less cells in the G1-phase and a higher number of cells in S-phase and G2/M transition (Fig. 4D). These data are in accordance with the reported function of PAK1 in mitotic progression [21].

### Phosphorylation-insensitive Function of Rac1

**Figure 5:**
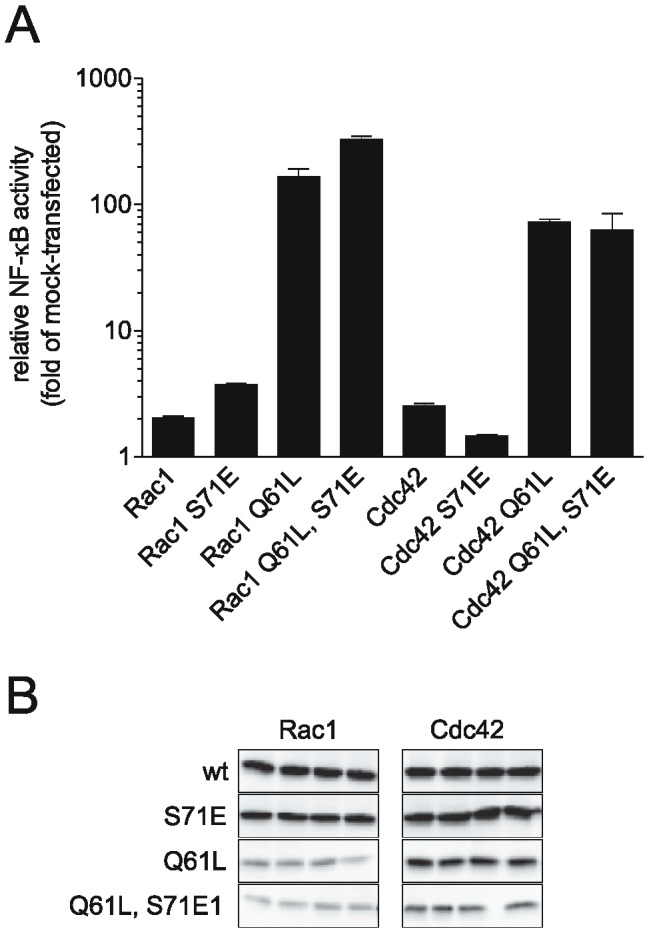
NF-κB is activated by phosphorylated Rac1. HEK 293 cells were co-transfected with NF-κB luciferase reporter plasmid as well as with Rac1 and Cdc42 mutants. Cells were lysed after 40 hours and analyzed for luciferase activity. Relative fold activity of mock-transfected cells is shown (arithmetic means±SD, n = 4). B) Expression of HA-tagged GTPases of quadruplicate samples from reporter gene assays was visualized by immunoblot using an anti-HA antibody.

Since Rac1 S71E showed active conformation as tested by PAK-PBD pull downs and bound to effector proteins IQGAP and MRCK, we investigated whether phosphomimetic Rac1 and Cdc42 are able to activate specific pathways. We choose the NF-κB pathway because the activation by Rac1 and Cdc42 is well described [22] and can easily be monitored by a reporter gene assay. Activation of the transcription factor NF-κB regulates the expression of genes required for inflammatory responses, cell growth and suppression of apoptosis. To measure the effect of Ser-71-phosphorylated Rac1 and Cdc42 on NF-κB activity, HEK-293 cells were transfected with corresponding Rac1 and Cdc42 expression vectors and a luciferase reporter plasmid containing three NF-κB-responsive sites. Fig. 5 shows the activation of the NF-κB-reporter after transfection of cells with the indicated GTPases. Expression of Rac1 induced two-fold and Rac1 S71E induced four-fold NF-κB-reporter gene activation compared to mock transfected cells. Constitutively active Rac1 Q61L and Rac1 Q61L/S71E were even more potent, leading to 200-fold and 300-fold activation, respectively. These results nicely demonstrate the ability of phosphomimetic Rac1 to activate effectors to the same magnitude as wildtype Rac1. Expression of Cdc42 and Cdc42 S71E as well as Cdc42 Q61L and Cdc42 Q61L/S71E showed activation of NF-κB that was in accordance to the set of Rac1 experiments. The expression level of the Cdc42 and Rac1 mutants was validated by immunoblots against HA-tag of ectopically expressed GTPases (Fig. 5B).

## Discussion

Rho GTPases are involved in various signaling pathways that regulate diverse cellular functions [23]. Especially Rac1 has diverse functions, but the predominant role of Rac1 is the regulation of actin-based processes such as membrane ruffling and lamellipodia formation or cell migration [24]. Rac1 is also involved in further actin-dependent processes like phagocytosis [25], [26]. In addition, Rac1 is part of signaling cascades, e.g. that of MAP kinases or NF-κB, that initiate gene expression, or in activation of the NADPH oxidase which results in production of reactive oxygen species [27], [28]. It is an unsolved question how activated Rac1 addresses specific pathways at a given time whilst being capable in principle of activating all of them simultaneously. In one theory, co-localization of Rac1 with specific effectors defines the functional outcome of Rac1 activation. Thus, the microenvironment might be decisive for Rac1 function. We here describe a potential, alternative regulatory mechanism, based on phosphorylation of Ser-71, by which Rac1 can be directed towards specific effectors, and which might also be applicable for Cdc42.

We previously reported that Ser-71 phosphorylation of Rac1/Cdc42 is not accompanied with general loss of function [16]. In the present study we clarified the outcome of Ser-71 phosphorylation of Rac1 and Cdc42 with respect to interaction with downstream effectors. One essential tool for investigating phosphorylation of Rac1 and Cdc42 is the phosphospecific antibody. To date, there is no phosphospecific antibody available that specifically recognizes pSer-71 Rac1 or pSer-71 Cdc42. Thus, it is not clear whether an observed effect accounts for Rac1 or Cdc42 phosphorylation. We took advantage of Rac1-deficient murine fibroblasts to show that EGF induces phosphorylation of Rac1 but not of Cdc42. The absence of Rac1 expression in these cells confirmed that anti-phosphospecific antibody staining observed in Rac1-expressing control cells mostly corresponded to phosphorylated Rac1, and not Cdc42. It is reasonable to assume that this is also true for other cell lines, although this remains to be experimentally validated. Focus of the present study is the characterization of phosphorylated Rac1 (S71) by using the phosphomimetic mutant. Cdc42 S71E was also applied since we cannot exclude Cdc42 phosphorylation. Thus, Cdc42 was additionally investigated in key experiments to emphazise the finding of phosphorylation as principal mechanism to modulate interaction with effector proteins which, theoretically might also apply for Cdc42. According to amino acid sequence, RhoG also possesses consensus sequence for Akt-mediated phosphorylation. However, since overexpression of Rac1 S71E induced the specific phenotype of cells, we assume that Rac1 is crucial for EGF-induced filopodia.

This hypothesis is substantiated by the predominant morphotype of cells with increased pSer-71 Rac1 showing massive formation of filopodia (see Fig. 1B) - a morphotype that is also seen in HEp2 cells stably expressing phosphomimetic Rac1 S71E. Fig. 1B also shows that formation of filopodia is less pronounced in Cdc42 S71E transfected cells compared to Rac1 S71E. Key experiments with transient expression of Rac1 S71E showed that formation of filopodia was not accompanied with increased activity of Cdc42. Due to cross talk between Cdc42 and Rac1 signaling a predominant Cdc42 morphotype can also be achieved by reducing Rac1 activity. For instance, filopodia formation downstream of Cdc42 could be enhanced by concomitant inhibition of Rac1 signaling [29], [30]. Furthermore, Cdc42-induced filopodia formation was described to be increased rather than decreased upon inhibition of Rac1 signaling to Arp2/3-mediated actin assembly through WAVE-complex. Dominant filopodia formation might thus very likely result from decreased signaling of Rac1S71E to WAVE and the Arp2/3-complex due to defective interaction with the WAVE-complex subunit Sra-1, as shown here by pull down experiments. Filopodia formation can also be induced by specific inactivation of Rac1 by *C. sordellii* Lethal Toxin TcsL-82 from strain IP82, which glucosylates Rac1 but not Cdc42 [31]. Thus, we interpret filopodia formation seen here predominantly resulting from loss of function of phosphorylated Rac1.

Besides Rac1, we also analyzed the effector binding properties of the phosphomimetic mutant of Cdc42 to check, whether phosphorylation at Ser-71 of these highly homologous GTPases might uncover a common mechanism for regulation. The two most important features of Rac1/Cdc42 phosphorylation were as follows: 1) Phosphorylation at Ser-71 only leads to partial functional inactivation of these GTPases, and 2) residual functional activity of pRac1 and pCdc42 appears to be harmonized with respect to interaction with here investigated effectors.

The question about activity status of Ser-71 phosphorylated GTPases resulted from different observations. Kwon and coworkers observed that GTP-binding was abrogated when serine-71 was exchanged to alanine and, thus, concluded that modification of this amino acid interferes with activation of Rac1 [15]. In line with that study, Ozaki and coworkers observed inhibitory effect of PI3K/Akt mediated serine phosphorylation of Rac1 on ROS production [32]. Although Ozaki and coworkers did not give direct evidence of Ser-71 phosphorylation, it can be assumed that Akt-mediated Rac1 phosphorylation observed in that study is with respect to inhibitory feature of phosphorylation consistent with our data. In contrast to this, our previous findings strongly suggest that phosphorylation does not necessarily lead to inactivation [16]. The present study dissects the functional outcome of Ser-71 phosphorylation. Whereas GTP-binding was slightly reduced in Rac1 S71E (and not affected in Cdc42 S71E) this GTPase is predominantly in its active form, as shown by pull down assays, membrane localization, and lack of Rho-GDI to reduce amount of pRac1 that can be precipitated in PAK-PBD pull down assay. Thus, functional inactivation appeared to mainly result from reduced effector interaction. Only specific effectors were sensitive towards non-phosphorylated Rac1/Cdc42 in our experiments. Here, interference with signaling was specifically restricted to the PAK1, Sra-1, or N-WASP. Other effectors, such as IQGAP1, 2 or MRCK alpha still bound to Rac1 S71E or Cdc42 S71E. We therefore conclude that phosphorylation at Ser-71 does not inactivate Rac1 and Cdc42, but restricts effector interaction. It has to be shown whether phosphorylation leads to loss of some specific differences between Cdc42 and Rac1 signaling in favour of a more harmonized signalling. Whereas induction of filopodia formation and lack of increased phosphorylation of PAK1/2 seen after Rac1 S71E expression was considered to be caused by loss of specific Rac1 functions, activation of the NF-κB signaling pathway by Rac1 S71E serves as example for positive signal transduction. In fact, latest results show positive interaction of Rac1 S71E with Nox1 and even increased interaction with NoxA1 (data not shown). These findings are in absolute accordance since NADPH oxidase and NF-κB mutually affect each other: Rac1 S71E which showed increased interaction with NoxA1 was more potent in activation of NF-κB than wildtype Rac1, even with constitutive active background. Whether Rac1/phospho-Rac1-dependent activation of NF-κB reporter gene assay depends on the NADPH oxidase or otherwise is under current investigation. Interestingly, all these results are also in accordance with signal transduction of Rac1b, which was described to activate NF-κB [33] but fails to interact with full length PAK1 [34]. We therefore hypothesize that phosphorylation of Rac1 at Ser-71 is an alternative and transient way of Rac1 modulation, which corresponds to Rac1b on the expression level.

The set of effectors investigated in this study is sufficient to exemplify the bivalent feature of pRac1 (S71) as discussed above. The interaction with other effectors, such as p67^phox^, phospolipase C-*β*2, and others has to be tested in separate studies to describe their binding and activation [35], [36]. For a comprehensive list of Rac effectors, see review by Bishop and Hall [37]. We assume Ser-71 phosphorylation of Rac1 as an additional mechanism to specify Rac1 signaling towards selected pathways.

## Materials and Methods

### Material and Antibodies

G418 was obtained from PAA, Germany. Monoclonal anti-pPAK1 (Ser144) was from abcam, UK. Polyclonal anti-pPAK1/2 (Thr423/Thr402), polyclonal anti-IQGAP1, polyclonal anti-N-WASP(30D10), polyclonal anti-PAK1, and monoclonal anti phospho-Rac1/Cdc42 (Ser71) were from Cell Signaling, Germany; monoclonal anti-IQGAP2 (Clone BB9), Upstate, MA, USA; polyclonal anti-MRCK alpha, Santa Cruz Biotechnology, CA, USA; anti-VASP, rhodamin-phalloidin; peroxidase-conjugated goat anti-mouse IgG and goat anti-rabbit IgG, Rockland, PA, USA; polyclonal Sra-1 antiserum was generously provided by Theresia Stradal (University of Münster,Germany), anti-HA (clone HA-7), and guanosine-5′-[γ-thio]triphosphate tetrasodium salt solution (GTP[γS]), Sigma, MO, USA; guanosine-diphosphate (GDP), human epidermal growth factor (EGF) was from R&D Systems, Germany; oligonucleotides were synthesized by Eurofins, Germany; Quickchange XL site-directed mutagenesis kit, Stratagene, Germany; Fugene 6 and Fugene HD transfection reagent, Roche, Germany; reporter lysis buffer, Promega, Australia; SuperSignal West Femto Substrate, Pierce, Rockford, USA; the construct for PAK-PBD domain (aa 56–272) was generously provided by John Collard (Netherlands). *Rac1^fl/fl^* fibroblasts were generously provided by Cord Brakebusch, Kopenhagen, Denmark.

### Expression of Recombinant Proteins

Exchange of serine for glutamate is an established method to mimic phosphoserine. Mutagenesis of Ser-71 to Glu-71 in Rac1 and Cdc42 was performed according to a standard protocol supplied with the site-directed mutagenesis kit Quickchange XL using 5`-GATTACGCCCCCTAGAATATC CGCAAACAGATGTG-3` (sense) and 5`-CACATCTGTTTGCGGATATTCTAGGGGGCGTA ATC-3′ (antisense) primer for Rac1 and 5`-GATTACGACCGCTGGAATATCCACAAACAGA TGTA-3` (sense) and 5`-TACATCTGTTTGTGGATATTCCAGCGGTCGTAATC-3′ (antisense) primer for Cdc42 amplification. The appropriate expression vectors (pGEX Rac1/Cdc42 and pcDNA3.1 3x HA Rac1/Cdc42) were used as template for PCR.

Likewise, the mutagenesis of Ser-71 to Ala-71 in Rac1 and Cdc42 was performed using 5`-GATTACGCCCCCTAGCCTATCCGCAAACAGATGTG-3` (sense) and 5`-CACATCTGTTTG CGGATAGGCTAGGGGGCGTAATC-3′ (antisense) primer for Rac1 and 5`-GATTACGACCG CTGGCTTATCCACAAACAGATGTA-3` (sense) and 5`-ACATCTGTTTGTGGATAAGCCA GCGGTCGTAATCT-3′ (antisense) primer for Cdc42 amplification. Mutagenesis of Gln-61 to Leu-61 in Rac1 and Cdc42 was performed using 5`-GGGATACAGCTGGACTAGAAGA TTATGACAGATT-3` (sense) and 5`-AATCTGTCTAATCTTCTAGTCCAGCTGTATCCC-3′ (antisense) primer for Rac1 and 5`-GATACTGCAGGGCTAGAGGATTATGACAGA-3` (sense) and 5`-TCTGTCATAATCCTCTAGCCCTGCAGTATC-3′ (antisense) primer for Cdc42 amplification. Rac1 S71E/Cdc42 S71E, Rac1 S71A/Cdc42 S71A and Rac1 Q61L/Cdc42 Q61L were expressed in E. coli after standard protocol for glutathione-S-transferase fusion proteins.

GTPases for fishing experiments were expressed in *Escherichia coli* and purified following standard procedures for glutathione-S-transferase fusion proteins. GST-GTPase fusion proteins bound to glutathione-beads were used for pull down experiments.

### GTP Binding Assay

Recombinant Rac1, Rac1 S71E, Cdc42 and Cdc42 S71E (2.5 µg of each) were incubated with 13,5 µCi [γ-^32^P]GTP in 700 µl binding buffer (50 mM Hepes pH 7.6, 0.2 mg/ml BSA and 0.5 mM EDTA) at 15°C for indicated times. The samples were then applied to a nitrocellulose membrane that had been rinsed with 3 times with 1 ml wash buffer. The filters were immediately washed 3 times with 1 ml of ice-cold wash buffer (50 mM Hepes pH 7.6, 150 mM NaCl and 10 mM MgCl_2_) and soaked in scintillation solution for 30 min. GTPase bound radioactivity was counted by liquid scintillation spectrometry.

### Cell Culture and Transfection

HEK293 cells were cultured in DMEM with sodium pyruvate (PAA, Germany), supplemented with 10% (v/v) fetal bovine serum, 100 U/ml penicilline, 100 µg/ml streptomycine at 37°C in humified air with 5% CO_2_ [38]. For transfection, cells were grown to subconfluence in 6 well plates and transfected with Fugene 6 transfection reagent according to manufactorer’s protocol. HEp2 cells were grown in MEM Eagle’s medium, supplemented with 10% (v/v) FBS, 100 U/ml penicilline and 100 µg/ml streptomycine at 37°C in humified air with 5% CO_2_ [16]. Stable transfected HEp2 cells were generated by transfection with pcDNA3.1 Rac1 or pcDNA3.1 Rac1 S71E constructs or with empty vector and cultured in the presence of 750 µg/ml G418 for six weeks. G418-resistant clones were selected and expression of HA-tagged Rac1/Rac1 S71E was monitored. After selection of positive clones further cell culture of stable transfected cells was performed in the presence of 400 µg/ml G418.

*Rac1^fl/fl^* fibroblasts were kindly provided by Cord Brakebusch and are homozygous for the loxP-flanked Rac1 allele described before [39]. Generation of *Rac^−/−^* fibroblasts will be described elsewhere. *Rac1^fl/fl^* and *Rac1^−/−^* fibroblasts were maintained in DMEM containing 10% (v/v) fetal bovine serum, 2 mM glutamine, 1 mM Na^+^ pyruvate and 1% non-essential amino acids (Invitrogen, Germany).

### Immunoblots

Cell lysates or precipitates from pull down assays were subjected to SDS-PAGE and subsequent transfer onto nitrocellulose. After blocking with Tris-buffered saline containing 3% (w/v) milk powder and 0.2% (v/v) Tween-20, the nitrocellulose membrane was incubated with the appropriate primary antibody diluted 1∶1.000 in TBS-T supplemented with 3% (w/v) bovine albumine over night at 4°C. The membranes were washed three times with TBS-T and incubated with the corresponding HRP-conjugated secondary antibody diluted 1∶5.000 in TBS-T for 30 min. Bound antibodies were visualized by incubation with SuperSignal West femto chemiluminescence substrate (Pierce).

### Flow Cytometry

Flow cytometry was performed to estimate number of cells within different cell cycle phases. The DNA content was measured using the fluorescent nucleotide acid dye propidium iodide. Therefore, cells were suspended by trypsinization and app. 5×10^5^ cells were fixed in ice cold ethanol (70%) for 30 min. After washing once with 1% bovine serum albumin in PBS, the total DNA content was stained with propidium iodide (150 µg/ml in Tris/HCl, pH 7.4, containing 1% BSA and 1% Triton X-100). RNA was removed by incubating cells with 0.5% RNase for 30 min. Subsequently, cells were subjected to FACS-analysis (FACScan flow cytometer, Becton Dickinson). A fluorescence area (FL2) of 400 was set to correlate with a 2n-set of chromosomes within the G1-phase. Cells found in the sub-G1-phase were considered as apoptotic and necrotic due to the decrease in DNA content.

### Western Blot Analysis

Protein samples were separated by SDS-PAGE and transferred onto nitrocellulose membrane. After blocking with 5% (w/v) nonfat dry milk in TBS-T (50 mM Tris HCl pH 7.2, 150 mM NaCl, 0.05% (v/v) Tween 20) the membrane was incubated overnight with the primary antibody at 4°C. After washing with TBS-T it was incubated for 45 min at room temperature with the appropriate horseradish peroxidase-conjugated secondary antibody. Detection was performed by means of enhanced chemiluminescence.

### Luciferase-based Reporter Assay

HEK-293 cells were transiently cotransfected with 50 ng of NF-κB luciferase reporter plasmid p3ENhκBcona-Luc containing three tandem repeats of NF-κB sites from the immunoglobulin Gκ promoter and 1 µg of the HA-tagged Rho GTPases. At 40 h post transfection, cells were washed with PBS and lysed in reporter lysis buffer. Luciferase activities were measured in cleared lysates with a luciferase assay system in accordance with manufacturer’s instructions. NF-κB activity was calculated as per cent of induction compared to that of pcDNA3.1_Cdc42 Q61L (constitutively active Cdc42) transfected cells.

### Pull Down Experiments

Pull down experiments with the PAK-PBD domain to detect active Rac1, Cdc42 or P-Ser-71 Rac1/Cdc42 were performed as previously described by Schoentaube [16]. Fishing experiments were either performed with constitutive active GTPases or with GDP/GTP-loaded GTPases. The nucleotide exchange of GTPases was performed at 30°C using 10 mM EDTA in 20 mM Tris pH 7.4 and 25 mM NaCl to extract bound nucleotide from the GTPase. After loading with either 1 mM GDP or 1 mM GTP[γS] for 15 min, the complex of GTPase and nucleotide was stabilized by addition of 50 mM MgCl_2_.

For pull down experiments HEp2 cells grown in 75 cm2 flasks were lysed in 2 ml ice-cold Fish-buffer (50 mM Tris pH 7.4, 2 mM MgCl_2_, 10% glycerine, 100 mM NaCl, 1% NP40, 0.5 mg/ml BSA). After 5 min incubation on ice the lysates were centrifugated at 16,000 g for 5 min. The supernatant was split into four samples of 0.5 ml each and used for precipitation of desired proteins in parallel to guarantee identical protein load in all samples. Therefore, 20 µl of bead slurry with bound GST-fusion protein of the respective bait (app. 15 µg each) were added to each sample and rotated at 4°C for 60 min. The beads were collected by centrifugation at 10.000 g and washed twice with Fish-buffer and subjected for SDS-PAGE.

### Cdc42 Activity Assay

The G-LISA assay from Cytoskeleton was used to assess active GTP bound Cdc42 in cell lysates. Subconfluent HEp2 cells were transfected with different Rac1 constructs. At 24 h post transfection the cells were analyzed according to manufacturer’s revised instructions. The level of activation was measured by reading the absorbance at 490 nm.

### MALDI-TOF/TOF Analysis

Washed precipitates from pull down experiments were separated by SDS-PAGE and stained with Coomassie brilliant blue. Specific bands were cut out, destained, digested with trypsin (12.5 ng/μl) and the generated peptides were extracted and solved in 10% acetonitril containing 0.2% trifluoroacetic acid. Peptides were cocrystalized with alpha-Cyano-4-hydroxycinnamic acid (4 mg/ml) in 50% acetonitril and 0.2% trifluoroacetic acid on an anchor target (Bruker Daltonic). After crystallization the peptides were analyzed in an MALDI-TOF/TOF mass spectrometer (Ultraflex I, Bruker Daltonic). Masses were determined by external calibration using suitable standard peptides (Bruker Daltonic). MS and MS/MS spectra were generated and analyzed with the BioTools (Bruker Daltonik) and MASCOT (Matrix Science, UK) software package.
